# Optical Micro/Nanofiber-Based Localized Surface Plasmon Resonance Biosensors: Fiber Diameter Dependence

**DOI:** 10.3390/s18103295

**Published:** 2018-09-30

**Authors:** Kaiwei Li, Wenchao Zhou, Shuwen Zeng

**Affiliations:** 1 Guangdong Key Laboratory of Optical Fiber Sensing and Communications, Institute of Photonics Technology, Jinan University, Guangzhou 510632, China; likaiwei11@163.com; 2 State Key Laboratory of Applied Optics, Changchun Institute of Optics, Fine Mechanics and Physics, Chinese Academy of Sciences, Changchun 130033, China; 3 XLIM Research Institute, UMR 7252 CNRS/University of Limoges, 87060 Limoges CEDEX, France

**Keywords:** localized surface plasmon resonance, fiber optic biosensor, optical micro/nanofiber

## Abstract

Integration of functional nanomaterials with optical micro/nanofibers (OMNFs) can bring about novel optical properties and provide a versatile platform for various sensing applications. OMNFs as the key element, however, have seldom been investigated. Here, we focus on the optimization of fiber diameter by taking micro/nanofiber-based localized surface plasmon resonance sensors as a model. We systematically study the dependence of fiber diameter on the sensing performance of such sensors. Both theoretical and experimental results show that, by reducing fiber diameter, the refractive index sensitivity can be significantly increased. Then, we demonstrate the biosensing capability of the optimized sensor for streptavidin detection and achieve a detection limit of 1 pg/mL. Furthermore, the proposed theoretical model is applicable to other nanomaterials and OMNF-based sensing schemes for performance optimization.

## 1. Introduction

In recent years, there is a growing need for fast, reliable, and highly sensitive testing systems in the field of environmental monitoring, food safety, drug development, biomedical research, and clinical diagnosis. Fiber optic sensors are promising candidates to fulfill the requirements, due to their merits of small footprint, compact size, fast response, low cost, and immunity to electromagnetic interference [1]. To render the fiber with special sensing abilities, as well as enhancing sensing performance, various functional materials are usually coated onto the fiber surface. These functional materials include nanoparticles [2,3,4,5,6,7,8], nanowires [9,10,11], nanofilms [12,13] and 2-D materials [14,15,16,17]. For example, by coating plasmonic nanoparticles on the surface of a tapered optical fiber, compact (localized surface plasmon resonance) LSPR biosensors [4,5,6,7] and (surface-enhanced Raman scattering) SERS chemical sensors [18] can be achieved. Optical fibers decorated with Pd nanoparticles [3] or Pd nanofilms [13] can serve as reliable H_2_ gas sensors. A coating of silica nanoparticles can make the fiber sensor suitable for humidity sensing [8]. Optical fiber sensors with graphene coatings are capable of ammonia gas detection [16,17]. Although optical fiber sensors with various nanomaterial coatings have been extensively studied for diversity applications, optical fibers themselves, as a major part of the sensor, are seldom studied and optimized. In this paper, we utilize gold nanoparticles as a model to study how the diameter of the optical micro/nanofiber (OMNF) influence the performance of the sensor. Our analysis shows that by optimizing the diameter of the OMNF, the refractive index (RI) sensitivity and the biosensing performance can be greatly improved.

Plasmonic sensors based on LSPR of noble metallic nanostructures have attracted the interest of many researchers over the past years. The sensing principle relies on the high sensitivity of the plasmonic resonant band of noble metal nanostructures for ambient dielectrics, which arises when the incident photon frequency is resonant with the collective oscillation of the conduction electrons in the metal structures [19,20]. Flat substrate-supported arrays of noble metal nanoparticles are one of the most versatile platforms for nanoplasmonic sensors [21]. However, the sensitivities of these sensors are rather limited, due to the small extinction cross-section of a single nanoparticle, and the relatively large area of a free space light beam.

Integrating plasmonic nanoparticles with optical fibers to enhance the light–matter interaction is a smart strategy to enhance the plasmonic sensing ability. This sensor scheme can realize remote sensing in narrow spaces with very small sample volumes, and even in vivo sensing. A few previous studies have demonstrated the feasibility of this optical fiber-based LSPR sensor for RI sensing and biosensing [22,23]. The RI sensitivities are on the order of several unites Δ*A*/RIU [22], and the limits of detection (LOD) are normally several tens of pM level [23]. Several studies tried to optimize the optical fiber geometry to expose more optical power into the evanescent field to enhance the photon-to-plasmon conversion efficiency, aiming at improving the sensitivity. These sensors include U-shaped optical fibers [24] and tapered optical fibers [6]. For example, Lin, et al. have tried to utilize a tapered optical fiber with a diameter of 45 µm as substrates for enhanced biosensing [6]. However, the optical fibers used are rather thick, making the fraction of optical power in the evanescent field rather small and, thus, limited the sensitivity. LSPR sensors based on OMNFs, with diameters close to or even below wavelength, are rarely reported.

One of the most important advantages of the OMNF is that it offers large fractions of evanescent fields and high surface field intensity, making it highly sensitive to disturbances in the surrounding medium [25,26]. The OMNF is an ideal platform for light-matter interactions. Thus, in this study, we combine the OMNF and gold nanoparticles to form an ultrasensitive nanoplasmonic sensor. Firstly, we build a theoretical model for the OMNF-based LSPR sensor and optimize the fiber diameter. Then, we experimentally demonstrate our theoretical results through RI sensing. We also show that our sensor can be easily integrated into a silicon chip array, which is promising for high throughput sensing applications. Further, we apply the sensor to biosensing and achieve a low LOD of 1 pg/mL.

## 2. The Principle of OMNF-Based LSPR Sensor

**Figure 1:**
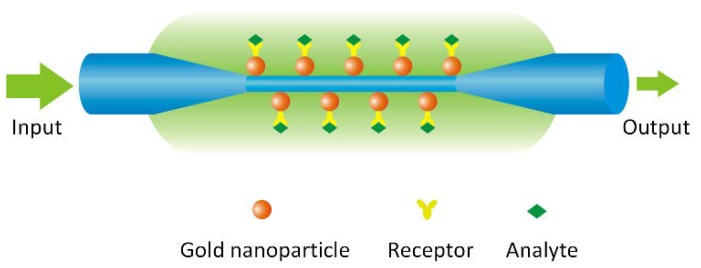
A schematic diagram showing the principle of optical micro/nanofiber (OMNF)-based localized surface plasmon resonance (LSPR) sensor.

The schematic diagram of the OMNF-based LSPR sensor is shown in Figure 1, where the OMNF works as the transducer, and the gold nanoparticles on the fiber surface as the sensing elements. The collective oscillation of the conductive electrons in the gold nanoparticles can be excited by the evanescent wave of the OMNF. It can detect subtle RI changes adjacent to the gold nanoparticles by monitoring the transmission spectra. Further, it can work as a biosensor by decorating the nanoparticles with specific receptors.

**Figure 2:**
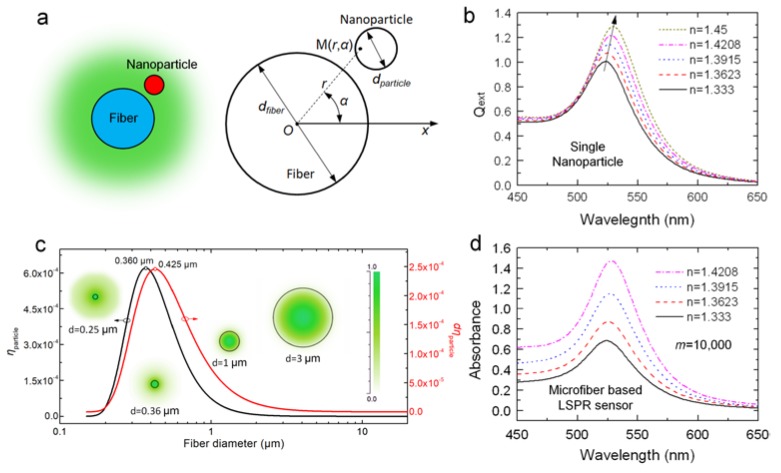
(**a**) Schematic diagram of the model; (**b**) Extinction of a 20 nm-sized gold nanoparticle with different surrounding refractive indexes (RIs); (**c**) Calculated $\eta_{\mathrm{particle}}$ and $d\eta_{\mathrm{particle}}$ of a 20 nm-sized nanoparticle located on the surface of a OMNF as a function of fiber diameter (wavelength: 520 nm); (**d**) Simulated spectral responses of a OMNF-based LSPR sensor to different surrounding RIs (*d*_fiber_ = 1 µm, *d*_particle_ = 20 nm, *m* = 10,000, wavelength: 520 nm).

First, we built an analytical model for the sensor. For adiabatic OMNFs, the optical power is mainly carried in the fundamental LP_01_ mode. Here, we consider the situation where the nanoparticles on the OMNF are isolated from each other, and interparticle coupling is negligible. This assumption is reasonable, due to the fact that only a small amount of particles can induce great optical loss to OMNFs, owing to their strong evanescent field. Suppose the number of nanoparticles on the fiber surface is *m*, the input optical power and the transmission of the bare microfiber are *P*_0_ and *T*_fiber_. Assign the output power and the absorption of the fiber after gold nanoparticles immobilization to be *P* and *A*, respectively. Then, we can get [27,28]

(1) $$P=P_{0}T_{\mathrm{fiber}}\exp\left(-mQ_{\mathrm{ext}}\eta_{\mathrm{particle}}\right),$$

(2) $$A=-\ln T_{\mathrm{fiber}}+mQ_{\mathrm{ext}}\eta_{\mathrm{particle}},$$

where *Q*_ext_ stands for the extinction efficient of the spherical nanoparticle, which is defined as the ratio of the extinction cross-section and the geometrical cross-section of the particle. It can be calculated through Mie theory [29]. *η*_particle_ denotes the proportional optical power occupied by the cross-section of the nanoparticle (Figure 2a), and can be calculated by the following integration over the cross-section of the nanoparticle:

(3) $$\eta_{\mathrm{particle}}\;=\;\iint_{S_{\mathrm{particle}}}\;\frac{p\left(r,\alpha \right)}{P_{\mathrm{fiber}}}rdrd\varphi ,$$

where *P*_fiber_ is the total power cast on the cross-section of the fiber, and p(r,α) is the local optical power density at a point *M* in the cross-section. Both p(r,α) and *P*_fiber_ can be obtained by numerically solving the Helmholtz equations for OMNFs [30]. Hence, we can obtain $\eta_{\mathrm{particle}}$ numerically.

As mentioned above, we mainly focus on the optimization of OMNF in our analysis. Hence, we take the 20 nm-sized gold nanosphere as a model, and the absorption of a single nanoparticle $Q_{\mathrm{ext}}\eta_{\mathrm{particle}}$ can be considered as constant for a certain surrounding RI (SRI). Thus, the output absorption is proportional to *m* and $\eta_{\mathrm{particle}}$. We calculate the extinction of the 20 nm-sized gold nanosphere with different SRI, and the results are shown in Figure 2b. The resonant peak rises gradually and redshifts as the SRI increases. This indicates that we can measure the changes of SRI through both wavelength modulation and intensity modulation. From Equations (1) and (2), it is clear that the sensing performance of OMNF-based LSPR sensor is a collective contribution of all the nanoparticles on the fiber surface. From this point of view, the sensitivity for SRI in wavelength modulation is only determined by the intrinsic property of gold nanoparticles. The number of gold nanoparticles can only influence the signal to noise ratio. Whereas for intensity modulation, both the coherent property of gold nanoparticles and the number of nanoparticles also contribute to the overall sensitivity. Therefore, we prefer the intensity modulation in our study.

For RI sensing, when the size and material of nanoparticles are determined, only the fiber diameter and the number of nanoparticles can determine the sensitivity. According to Equation (2), the absorption can be improved by optimizing the fiber diameter (*d*) or simply increasing the number of nanoparticles (*m*). The SRI sensitivity can be expressed as dA/dn. The output absorption *A* is a collective contribution of every nanoparticle on the OMNF. Thus, when SRI changes with a small value dn, a larger $\eta_{\mathrm{particle}}$ or a larger *m* will induce a larger change dA to the output absorption *A* and, hence, lead to a higher sensitivity.

For biosensing, only the nanoparticles with molecular binding even have local RI changes. In this case, $Q_{\mathrm{ext}}\cdot \eta_{\mathrm{particle}}$ essentially reflects the sensitivity. For a certain nanoparticle on the OMNF, the more optical power it encounters, the more it contributes to the output signal when the same number of biomolecules bind to the nanoparticle surface. Therefore, fiber diameter plays a dominant role in sensitivity. By optimizing the fiber diameter, we can maximize the fiber sensitivity. The number of nanoparticles determines both sensitivity and the dynamic response range of the sensor. The more particles on the sensor surface, the larger the dynamic response range is.

If we can assume the surface distribution density (*ρ*) to be the same for fibers with different diameters, thus, the number of nanoparticles on the fiber surface will increase as the fiber diameter increases. Assign the length of the OMNF as *L*. Then, we can get

(4) m=ρπdL.

Assume the tapers of the tapered optical fiber are adiabatic, then we can get $T_{\mathrm{fiber}}=1$. Hence, the absorption *A* can be further expressed as

(5) $$A=\pi \rho dLQ_{\mathrm{ext}}\eta_{\mathrm{particle}}.$$

Since *ρ*, *L*, and $Q_{\mathrm{ext}}$ are the same for the fibers with different diameters, the absorption *A* is determined by term $d\eta_{\mathrm{particle}}$.

Then, we calculate $\eta_{\mathrm{particle}}$ using our model (SRI = 1.333, $\eta_{\mathrm{fiber}}$ = 1.4513, wavelength: 525 nm). The results are depicted in Figure 2c. It is obvious that when fiber diameter decreases from 10 μm to submicron, $\eta_{\mathrm{particle}}$ dramatically improves and reaches a peak value at *d* = 0.36 μm. The modal field distribution in the inset clearly shows this tendency. However, when it falls below 0.36 μm, although the field intensity around the surface is still pronounced, the penetration depth of evanescent wave also increases dramatically, and more guided optical power transforms into an evanescent field. Thus, the proportional optical power near fiber surfaces is relatively reduced. This adequately explains the drop in $\eta_{\mathrm{particle}}$ when fiber diameter is smaller than 0.36 μm. We further calculate the term $d\eta_{\mathrm{particle}}$ to include the influence of the fiber diameter on the number of particles, as we assume the surface distribution to be constant for fibers treated with the same procedures. As shown in Figure 2c, the optimal value of diameter shifts to 0.425 µm. This is because the fibers with larger diameters have more nanoparticles on the surface. These theoretical results provide a guideline for designing OMNF-based LSPR sensor. To summarize, the performance of fiber-based LSPR sensors for both RI sensing and biosensing can be greatly enhanced when fiber diameter decreases towards submicron.

With this model, we simulate the spectral response of an OMNF-based LSPR sensor to SRI (*d*_fiber_ = 1 µm, *d*_particle_ = 20 nm, *m* = 10,000, wavelength: 520 nm). As shown in Figure 2d, as the SRI increases from 1.333 to 1.4208, the absorbance of the sensor increases dramatically across the broad wavelength range of 450–650 nm, whereas the wavelength shift is difficult to distinguish. This indicates that the intensity modulation is more preferable than the wavelength modulation.

## 3. Materials and Methods

### 3.1. Materials

Standard SMF-28e single mode optical fiber (8.2/125 μm) was purchased from Corning Inc. (New York, NY, USA). Tetrachloroaurate (HAuCl_4_) and hydrofluoric acid (HF, 40%) were bought from Sinopharm Chemical Reagents Co., Ltd. (Shanghai, China). 3-Aminopropyl-triethoxysilane (APTES, 99%), glutaraldehyde (Grade II, 25% in H_2_O), phosphate buffer saline (PBS), and biotinylated bovine serum albumin (biotin-BSA) and streptavidin were purchased from Bioss (Beijing, China). Polydimethylsiloxane (PDMS) was purchased from Dow Corning (Midland, MI, USA). All the reagents were of analytical grade. All solutions were prepared using deionized water (18.2 MΩ. cm) obtained from a Milli-Q filtration system (Millipore, Burlington, MA, USA).

### 3.2. Experimental Setup

**Figure 3:**
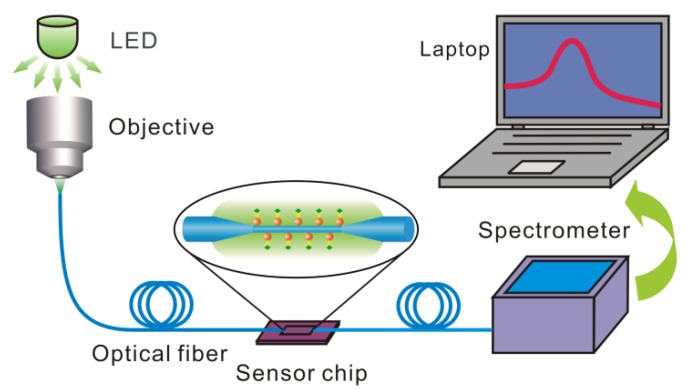
The schematic diagram of the experimental setup.

Figure 3 depicts the experimental setup, in which a LED or halogen lamp is used as a broadband light source. The light is coupled into the optical fiber by an objective lens. The OMNF is fixed in a silicon fluid cell and the cell is sealed with a PDMS cover to form a chamber for the delivery of sample solutions. The transmitted light is then collected by a miniature spectrometer and the data are acquired by a laptop.

### 3.3. Fabrication of OMNFs

Normally, OMNFs can be fabricated via two methods, one is the heating and drawing method [31], another one is the chemical etching method [27,28]. The former method features fast fabrication and high surface quality. However, efficiently transferring the OMNFs from the drawing apparatus to microfluidic chips remains problematic because the OMNF is very fragile. Here, we choose the chemical etching method. Although this method is time-consuming and, to some extent, dangerous, it is easier for realizing mass production. More importantly, the etching process can be accomplished by an on-chip method, thus avoiding the need to transfer the OMNFs, and greatly improved the success rate.

**Figure 4:**
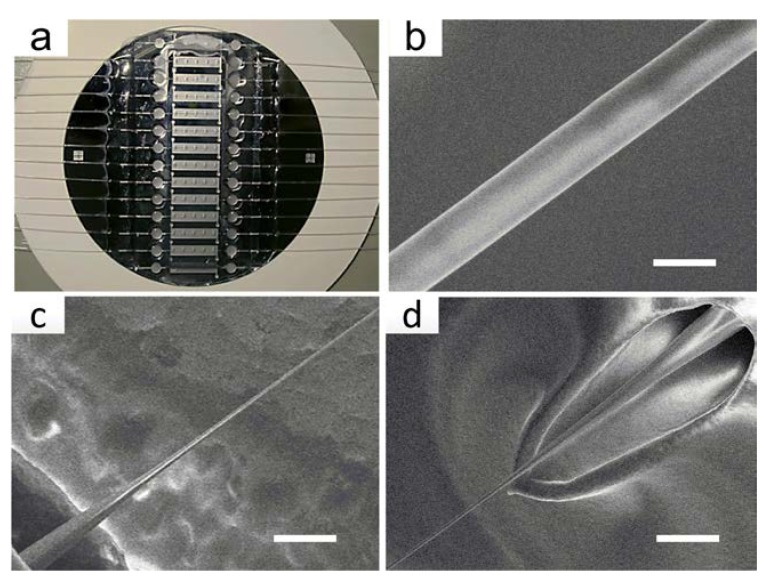
Fabrication of the OMNF. (**a**) OMNF sensor arrays; (**b**) An scanning electron microscope image showing the uniform waist of the fabricated OMNF (scale bar: 1 µm); (**c**,**d**) Down-taper and up-taper of the OMNF (scale bar: 10 µm).

Typically, a segment of 10 mm in the middle of a single-mode optical fiber is stripped off the coating jacket and cleaned with acetone. Then, the bare fiber is fixed in the fluid cell by PDMS. Afterward, we add etching solution into the fluid cell, and the etching process is monitored by an online monitoring system. The fluid cells are fabricated on silicon chips through MEMS technology, and a typical image is shown in Figure 4a. A typical OMNF with a diameter of 1.0 μm and the transition tapered regions are shown in Figure 4b–d. The fabricated OMNF have a waist length of about 6.0 mm.

### 3.4. Gold Nanoparticle Synthesis

The gold nanoparticles are synthesized by a method introduced by Turkevich [32]. The sizes of the gold nanoparticels are confirmed by scanning electron microscope images, and the UV-VIS absorption spectrum is also measured by a Lambda 850 UV-VIS spectrophotometer. The mole concentrations of the gold nanoparticle solutions are calculated according to the equation proposed by Haiss [33].

### 3.5. Fiber Surface Functionalization and Gold Nanoparticle Decoration

Briefly, the as-fabricated OMNF is immersed in a bath consisting of 1 vol of 30% H_2_O_2_ and 3 vol of concentrated H_2_SO_4_ for 10 min to generate reactive hydroxyl groups. The cleaned fiber surface is then immersed in a 5% solution of APTES in acetone for 2 min, and then thoroughly washed with acetone for 6 times and DI water for 30 min, sequentially [28]. After silanization, the surface of the OMNF is covered with a layer of amino groups, and shows a positive charge. Then, the gold nanoparticle solution is added, and the nanoparticles bind to fiber through electrostatic force. The solution is replaced with DI water once the adsorption reaches saturation.

### 3.6. Receptor Immobilization and Blocking

In order to endow the OMNF-based LSRP sensor with biospecificity, we need to decorate the gold nanoparticle with recognition receptors, such as antibodies and aptamers. Here, we employed the classic binding pair of biotin and streptavidin, which have a strong specific affinity towards each other, as a model. First, we treat the fabricated sensor with 20 mM cysteine/water solution and incubated for 2 h. Then, we the immersed the sensor into glutaraldehyde water solution with a concentration of 2.5% for 2 h, and thoroughly rinsed with deionized water afterwards. Glutaraldehyde acts to add crosslinks between cysteine and the receptors. Then, we immobilize biotinylated BSA onto the gold nanoparticles by sinking the sensor into a biotinylated BSA solution with a concentration of 10 μg/mL for 2 h. Finally, the unreacted groups on the surface are blocked with BSA molecules by immersing the sensor in a BSA/PBS solution with a concentration of 10 mg/mL for 2 h.

## 4. Results and Discussion

### 4.1. Refractive Index Sensing

**Figure 5:**
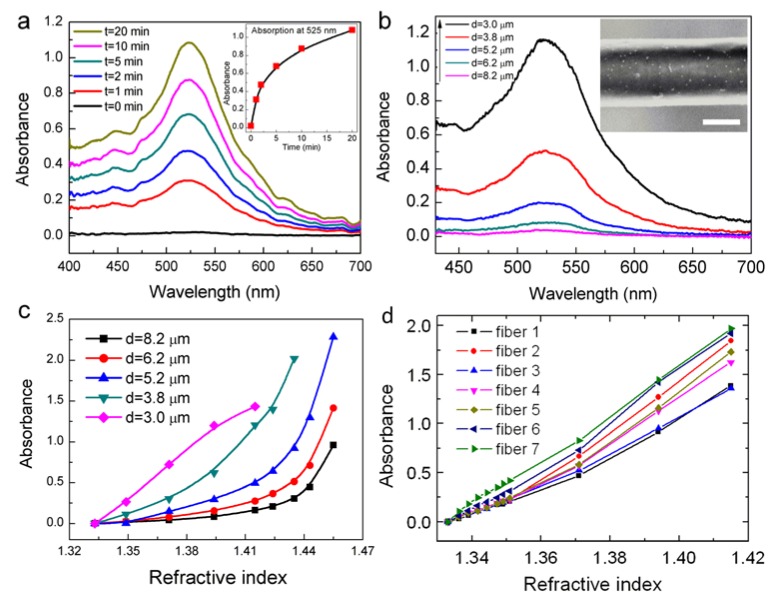
(**a**) Evolution of the output spectra of OMNF as gold nanoparticles bind to the fiber (inset: the evolution of absorbance at 525 nm); (**b**) Final absorption spectra of OMNFs with different diameters as gold nanoparticle binding reaches saturation; (**c**) RI sensing performances of OMNF-based LSPR sensors with different fiber diameters; (**d**) RI sensing performances of seven OMNF-based LSPR sensors with diameters of ~1 µm.

The influence of the fiber diameter on the RI sensitivity is experimentally verified. Figure 5a shows the evolution of the absorption spectrum during the immobilization process of gold nanoparticles (d = 8.2 μm). OMNFs with diameters of 1.0, 2.1, 3.0, 3.8, 5.2, 6.2, and 8.2 μm, are decorated with gold nanoparticles under the same conditions. The absorption spectra of the microfiber-based LSPR sensors with diameters of 3.0–8.2 μm are shown in Figure 5b (the spectra for OMNFs of 2.1 μm and 1.0 μm are not shown because the optical power is entirely depleted upon the adsorption of gold nanoparticles.). We can clearly see that the absorbance at wavelengths of 410–700 nm increase dramatically when the fiber diameter gets smaller. Considering the experimental parameters for gold nanoparticle decoration is the same for these sensors, it is reasonable to assume the surface density of the gold nanoparticles on the fiber surface is similar. Thus, there are fewer nanoparticles on thinner fibers. Therefore, we can come to a conclusion that the light and gold nanoparticle interaction can be greatly enhanced when the fiber diameter gets smaller, which agrees well with our theoretical predictions. The RI sensing is carried out by injecting glycerin/water solutions with different RIs. As shown in Figure 5c, LSPR sensors with thinner fiber diameter have higher sensitivity, and have good linearity in the low RI range. We can infer that LSPR sensor with a diameter of 1 μm could be even more sensitive. This is in good agreement with our theoretical predictions.

Here, we regard 1 μm to be the optimal diameter for OMNF-based LSPR sensors by comprehensively considering the sensitivity, fabrication limitations, as well as the mechanical robustness of the microfiber. Prior to the biosensing applications, we first systematically characterize the RI sensing characterization and reproducibility of the OMNF-based LSPR sensors with diameters of 1 μm. The results are displayed in Figure 5d, which indicate that the OMNF-based LSPR sensor exhibits good linear responses to the SRI and high reproducibility.

### 4.2. Biosensing

**Figure 6:**
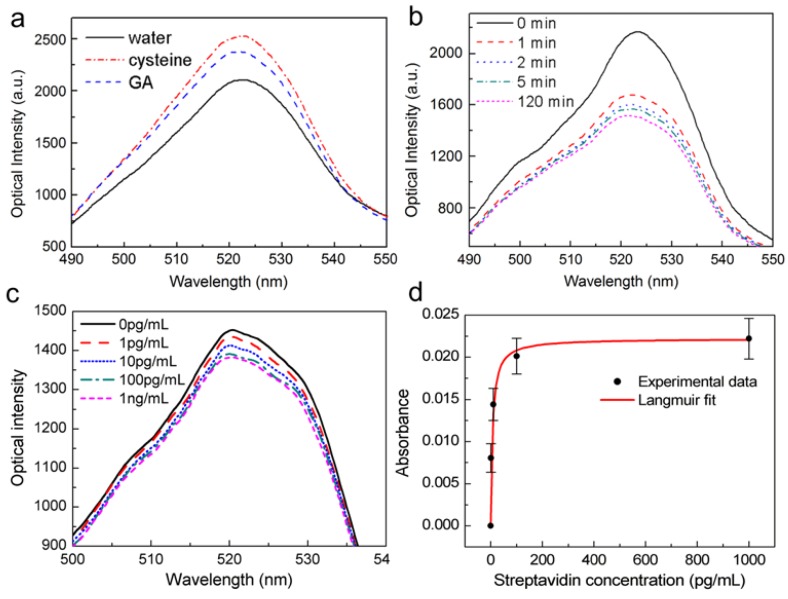
Spectral responses (**a**) after each step of gold nanoparticle surface functionalization; (**b**) during the immobilization process of biotinylated BSA; (**c**) to streptavidin with different concentrations; (**d**) Experimental results and Langmuir fit.

To demonstrate the biosensing ability of the proposed OMNF-based LSPR sensor, we use a biotin–streptavidin model as the receptor and target molecule. The output spectra of the OMNF-based LSPR sensor for each step of surface functionalization are recorded in Figure 6a. The output optical power increased after the cysteine treatment, indicating that the effective RI of the cysteine layer is lower than that of the citrate layer. After the crosslinker glutaraldehyde was added, a glutaraldehyde layer was formed on top of the cysteine layer, and the optical intensity dropped a little. Prior to receptor immobilization, the DI water in the sensor cell was replaced by PBS, and a baseline spectrum was measured. Then, the biotinylated BSA in PBS buffer solution with a concentration of 10 μg/mL was injected. The solution was left to settle for 2 h to allow the receptor molecules to fully adsorb to the gold nanoparticle surface. Figure 6b shows a typical example of the optical output spectrum during different stages of the biotinylated BSA immobilization. A rapid decline in the output optical power occurs in the first 2 min, followed by a much slower decrease. The decrease finally reached a plateau which indicates that the gold nanoparticle surface was fully covered by the biotinylated BSA molecules. Afterward, BSA solution with a concentration of 10 mg/mL in PBS buffer was added to block the unreacted sites on gold nanoparticle surface, and rinsed with PBS thoroughly.

The OMNF-based LSPR sensor decorated with biotin-BSA was then used for streptavidin detection. Different concentrations of streptavidin in PBS buffer were tested sequentially. The reaction time for each sample was 30 min, to make sure the binding of streptavidin to immobilized biotin reached saturation. Afterward, the sensor was thoroughly washed with PBS to eliminate the influence brought about by the small RI differences among the streptavidin solution samples. Figure 6c shows the optical spectral response of the OMNF-based LSPR sensor to different concentrations of streptavidin. The output optical intensity decreases with the increasing concentration of the streptavidin solution. This indicates that the amount of streptavidin on the immobilized gold nanoparticle surface increases as the concentration increases, thus forming a high RI biolayer around the gold nanoparticle. The presence of the high RI coating improves the optical extinction ability of the gold nanoparticles, causing an optical loss in the output spectrum.

To ensure the reproducibility, the streptavidin detection experiments were repeated 3 times using different sensors with similar parameters. The normalized results are summarized in Figure 6d. The errors bars represent the standard deviation of the measurement results at each concentration, and confirm the good reproducibility of the sensor. A possible reason for the variation is the manufacturing errors of the OMNFs. This fabrication error can also be observed in the RI sensing. Despite this, a LOD of about 1 pg/mL can be clearly distinguished from the results. Compared to previously reported fiber-based LSPR sensors that employ relatively thick optical fibers [23,34], our sensor is much more sensitive, and the LOD is at least 4 orders of magnitude lower. Although our sensor is higher in sensitivity, the linear dynamic range is relatively narrow, which may become a limitation in practical applications. A possible method to broaden the dynamic range is to increase the number of binding sites on the sensor surface via increasing the number of gold nanoparticles.

Finally, we use the Langmuir isotherm model (Equation (6)) to describe the reaction system where streptavidin molecules bind to immobilized biotin, since the output optical signal scales with the surface coverage [35].

(6) $$\Delta =\frac{\Delta_{\max}K_{a}C}{K_{a}+C},$$

where Δ is the instantaneous response, Δ_max_ is the saturation response, *C* is the protein concentration, and *K*_a_ is the Langmuir equilibrium constant of association [35]. A Langmuir fit was also plotted in Figure 6d, which follows the absorbance response to different concentrations of streptavidin well. An association constant *K*_a_ = 9.2 × 10^13^ M was obtained, which agrees well with published results for streptavidin and biotin bindings [36]. The ultrahigh association constant also confirms the specific binding between streptavidin and biotin binding in our sensing experiments.

## 5. Conclusions

We report a highly sensitive OMNF-based LSPR sensor. Localized surface plasmons are excited by the enhanced evanescent field of the OMNF. First, we built a theoretical model for the sensor and numerically studied the influence of the OMNF diameter on the sensing performance. Results show that by reducing the OMNF diameter, the photon to plasmon ratio could be greatly enhanced, which will, in turn, increase the sensitivity. The photon to plasmon ratio approaches a maximum value when the diameter is decreased to 0.36 μm. This is the theoretical optimum diameter for OMNF-based LSPR sensors. By further reducing the diameter, the photon to plasmon ratio begins to drop rapidly. Experiments were conducted to examine the impact on OMNF diameter on RI sensing performance. Results show that the sensitivity could be improved by several orders when the diameter was reduced from 8.2 μm to 1.0 μm. Further, we demonstrated the biosensing ability of the sensor using an OMNF of 1.0 μm to obtain both high sensitivity and good mechanical robustness. A LOD of 1 pg/mL was achieved with good repeatability. The ultra-high sensitivity makes this sensor suitable for small molecule detection and trace element assays.
